# Genome-Wide Analysis of Cotton miRNAs During Whitefly Infestation Offers New Insights into Plant-Herbivore Interaction

**DOI:** 10.3390/ijms20215357

**Published:** 2019-10-28

**Authors:** Jianying Li, J. Joe Hull, Sijia Liang, Qiongqiong Wang, Luo Chen, Qinghua Zhang, Maojun Wang, Shahid Mansoor, Xianlong Zhang, Shuangxia Jin

**Affiliations:** 1 National Key Laboratory of Crop Genetic Improvement, Huazhong Agricultural University, Wuhan 430070, China; lijy90@126.com (J.L.); sijialiang@webmail.hzau.edu.cn (S.L.); wangqq0515@163.com (Q.W.); chenluo@webmail.hzau.edu.cn (L.C.); qhzhang@outlook.com (Q.Z.); mjwang@mail.hzau.edu.cn (M.W.); xlzhang@mail.hzau.edu.cn (X.Z.); 2 USDA-ARS, Arid Land Agricultural Research Center, 21881 North Cardon Lane, Maricopa, AZ 85138, USA; joe.hull@ars.usda.gov; 3 National Institute for Biotechnology and Genetic Engineering (NIBGE), Faisalabad 38000, Pakistan; shahidmansoor7@gmail.com

**Keywords:** cotton, whitefly, insect resistance, miRNA, lincRNA, phasiRNA

## Abstract

Although the regulatory function of miRNAs and their targets have been characterized in model plants, a possible underlying role in the cotton response to herbivore infestation has not been determined. To investigate this, we performed small RNA and degradome sequencing between resistant and susceptible cotton cultivar following infestation with the generalist herbivore whitefly. In total, the 260 miRNA families and 241 targets were identified. Quantitative-PCR analysis revealed that several miRNAs and their corresponding targets exhibited dynamic spatio-temporal expression patterns. Moreover, 17 miRNA precursors were generated from 29 long intergenic non-coding RNA (lincRNA) transcripts. The genome-wide analysis also led to the identification of 85 phased small interfering RNA (phasiRNA) loci. Among these, nine *PHAS* genes were triggered by miR167, miR390, miR482a, and two novel miRNAs, including those encoding a leucine-rich repeat (LRR) disease resistance protein, an auxin response factor (ARF) and MYB transcription factors. Through combined modeling and experimental data, we explored and expanded the *miR390-tasiARF* cascade during the cotton response to whitefly. Virus-induced gene silencing (VIGS) of *ARF8* from miR390 target in whitefly-resistant cotton plants increased auxin and jasmonic acid (JA) accumulation, resulting in increased tolerance to whitefly infestation. These results highlight the provides a useful transcriptomic resource for plant-herbivore interaction.

## 1. Introduction

Cotton (*Gossypium spp.*) is a widely cultivated economic crop utilized for its fiber and oil-yielding capabilities that are negatively impacted by both abiotic and biotic stresses. *G. hirsutum* is an allotetraploid species (A_t_A_t_D_t_D_t_, 2n = 4× = 52, (AD)_1_ genome) comprising A_t_- and D_t_-subgenome that originated from the two diploid cotton species *G. raimondii* (D_5_) and *G. arboreum* (A_2_) [1]. Although transgenic cotton expressing *Bacillus thuringiensis* (*Bt*) cry toxins have been used successfully to control lepidopteran pests, including *Helicoverpa armigera* and *Pectinophora gossypiella*, similar technology has yet to be fully developed for activity against phloem-feeding pests, such as whitefly, aphid, and leafhopper [2,3,4]. Whitefly (*Bemisia tabaci*) is a destructive pest of many agronomically important crops, such as tobacco, tomato, lettuce and cotton, and causes extensive crop damage by directly sucking phloem sap and vectoring diverse viruses [5]. Recently, Shukla et al. (2016) identified a candidate protein from edible fern (*T. macrodonta*) that was toxic to whitefly [6]. Zhu et al. previously assessed the susceptibility of 400 elite cotton lines to whitefly infestation and identified 42 lines between resistance and the susceptibility spectrum [7].

A major portion of the eukaryote genome consists of non-coding sequences that were previously regarded as junk DNA. Non-coding RNAs (ncRNAs) are a group of regulatory molecules that fall into the following two major classes, long ncRNAs (lncRNAs), which are > 200 bp, and small ncRNAs (18–26 nt in length), which can be further divided into microRNAs (miRNA) and short interfering RNAs (siRNA). Primary miRNA transcripts are formed as miRNA precursors (pre-miRNA) from lncRNAs possessing a stem-loop structure. The *DICER-LIKE 1* (*DCL1*) enzyme processes the pre-miRNAs to form miRNA/miRNA^*^duplexes that are then assembled into the RNA-induced silencing complex (RISC) [8,9]. The mature miRNAs (20–24 nt) subsequently promote the cleavage of target mRNA sequences through base pairing. To date, intensive studies have been performed to examine miRNA targeting of protein-coding genes, though research on endogenous target mimics (eTM) is less common. Consequently, it is becoming increasing clear that small RNAs play important roles in suppressing, the expression of diverse target genes, both transcriptionally and post-transcriptionally, including those involved in the development, abiotic stress responses, and plant disease resistance [10,11,12].

miRNAs trigger the formation of a specialized class of phasiRNAs called trans-acting siRNA (tasiRNA) from non-coding *TAS* genes [13]. Double-strand RNAs (dsRNAs) generate tasiRNAs from miR173-, miR390-, and miR828-cleaved target transcripts from *TAS1/2*, *TAS3* and *TAS4* [14,15]. The generation of these miRNA-mediated tasiRNAs requires the involvement of *RNA-dependent RNA polymerase 6* (*RDR6*), which produces dsRNAs and 21nt secondary siRNAs that are subsequently cleaved by *DICER-LIKE 4* (*DCL4*). To date, approximately 3300 *PHAS* loci have been identified in 23 plant species, with most exhibiting distinct expression profiles that are associated with particular cellular conditions such as developmental stage, biotic stress (e.g., viral infection), and abiotic stress [16]. In addition to non-coding *PHAS* loci, some *PHAS* loci have also been identified in the protein coding regions of nucleotide-binding (NB), leucine-rich repeat (LRR), and pentatricopeptide repeat (PPR) proteins as well as the MYB transcription factor, all of which are encoded by disease resistance-related genes [17].

Plant-insect/pathogen interaction has forced plants to evolve elaborate defense systems, including non-host resistance via physical barriers, pathogen-associated molecular pattern (PAMP)-triggered immunity (PTI), and effector-triggered immunity (ETI) [18]. Plant responses to herbivore infestation are regulated by the interaction of diverse phytohormones such as jasmonic acid (JA), salicylic acid (SA), ethylene (ET), auxin and abscisic acid (ABA) [19]. JA can induce plant defenses through activation of lipoxygenase (LOX), 12-oxo-phytodienoic acid (OPDA) and cytochrome P450 expression during the susceptible plant-herbivory interaction [20]. The only known NB-LRR disease resistance genes (R genes) recognize pathogen/microbial effectors and play important roles in defense or symbiotic plant-pathogen/herbivore interactions [21,22].

Small RNAs (sRNAs) play critical roles in plant herbivore stress response by regulating the expression of diverse downstream genes. Among plant-herbivore interactions, a previous study reported on the expression profile of miRNAs and phasiRNAs in tobacco plants (*Nicotiana attenuata*) following infestation with tobacco hornworm (*Manduca sexta*). That study revealed that miRNAs were involved in both JA-dependent and JA-independent plant defense response signaling pathways [23]. Silencing of *RNA-DIRECTED RNA POLYMERASE1* (*RDR1*) in *Nicotiana attenuata* induced target gene expression and reduced JA levels, which enhanced ethylene biosynthesis and increased plant susceptibility to chewing herbivores [24]. Among plant and phloem-sucking insect interactions, aphid-induced miRNA expression profiles differed between *Vat^+^* (R gene for virus aphid transmission) and *Vat^-^* melons, suggesting that miRNAs mediate the auxin insensitivity phenotype in susceptible plants [25].

Here, we expand on our transcriptome assemblies to investigate the potential functions of non-coding RNAs, a whitefly-resistant cultivar (HR) and a susceptible cotton cultivar (ZS) response to whitefly infestation [26]. We, therefore, aimed to determine whether miRNA-mediated gene silencing plays a role in the cotton response to whitefly infestation. Degradome sequencing was used to identify miRNA targets and *PHAS* loci. We used gene enrichment analysis to examine the role of cotton miRNAs in modulating phytohormone signaling and focused on the miR390-targeted *ARF8* using VIGS. The current report illustrates the miRNA’s intricate transcriptional network in cotton plants in response to herbivore infestation.

## 2. Results

### 2.1. Classification and Annotation of sRNAs in Resistant (HR) and Susceptible (ZS) Cotton Cultivar in Response to Whitefly Infestation

The HR and ZS cultivars exhibited highly difference resistance after one month in response to whitefly infestation (Figure S1). To explore the relationship between miRNA and siRNA abundance and the different cotton cultivars’ responses to whitefly infestation, we constructed sRNA libraries with three biological replicates per sample type from the HR and ZS plants infested with adult whiteflies for 24 h and mock infested plants (Table 1). In total, 201,876,882 raw reads and 20,239,660 unique reads were generated from the twelve libraries. After discarding the adapter sequences, low quality reads, and short reads, the unique sRNAs were annotated using the Rfam database to exclude snoRNA, snRNA, and rRNA sequences (Table 1).

**Figure 1:**
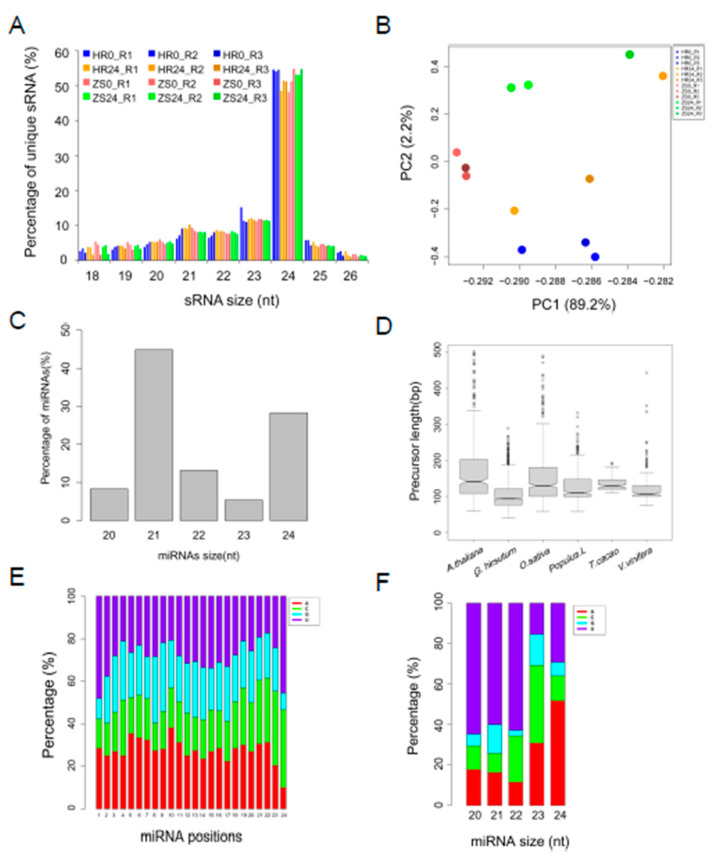
Identification and characterization of cotton miRNAs after whitefly infestation. (**A**) Distribution of unique sRNA length present in the three biological replicates. (**B**) Principle component analysis (PCA) of common sRNAs RPM from the twelve libraries. (**C**) Distribution of miRNA length. (**D**) Distribution of miRNA precursor length in cotton compared to four representative plant species. (**E**) Nucleotide preference at each position of the predicted miRNA. (**F**) Analysis of first nucleotide bias in the different miRNAs.

Analysis of the remaining data showed that approximately 80% of the siRNAs and miRNAs aligned the *G. hirsutum* acc TM-1 reference genome, with most reads ranging from 18 to 26nt in length (Figure S2), though the majority (> 70%) of the unique reads were between 21 and 24nt (Figure 1A). To provide an overview of the sRNA landscape, the common sRNAs were estimated from the 12 libraries. Principal component analysis (PCA) was performed using the common sRNA read per million (RPM) values (Figure 1B). Replicates in HR0 and ZS0 were cohesively clustered on the first two components, and the treatment and mock groups have distinct clusters. Additionally, the RPM values for the common unique reads from the two biological replicates were calculated by Pearson’s correlation coefficient (PCC), which showed a high correlation (PCC = 0.99) in all the biological replicate groups (Figure S2). Based on these analyses, the different replication groups have higher correlations, and the mock/treatment of HR/ZS samples was well separated, suggesting that our experiments were reproducible, and that the data obtained were reliable.

In total, 475 miRNA precursors were identified, including 260 unique miRNA sequences consisting of 110 conserved and 150 novel miRNAs from the twelve sRNA libraries (Table S2). The length of the miRNAs ranged from 20 to 24nt, with 21nt miRNAs predominating (Figure 1C). Comparing the miRNA precursor gene length identified in this report with those from four other representative plant species, it was found that cotton miRNA precursors were shorter, with a median size < 100nt (Figure 1D). The nucleotide bias at each position was also analyzed to better understand the miRNA cleavage sites [27]. In general, motifs that could be defined as miRNA cleavage sites lacked the nucleotide uracil (U) at the 4^th^ position, whereas the 10^th^ position tended to be adenine (A) (Figure 1E). We also found that the first position in the 21nt miRNAs was predominantly U but was A in the 24nt miRNAs (Figure 1F). A portion (110 of 260) of the unique miRNA sequences identified corresponded to the 46 miRNA families that currently comprise the miRBase21 database, those sequences were therefore defined as “conserved miRNAs”.

**Figure 2:**
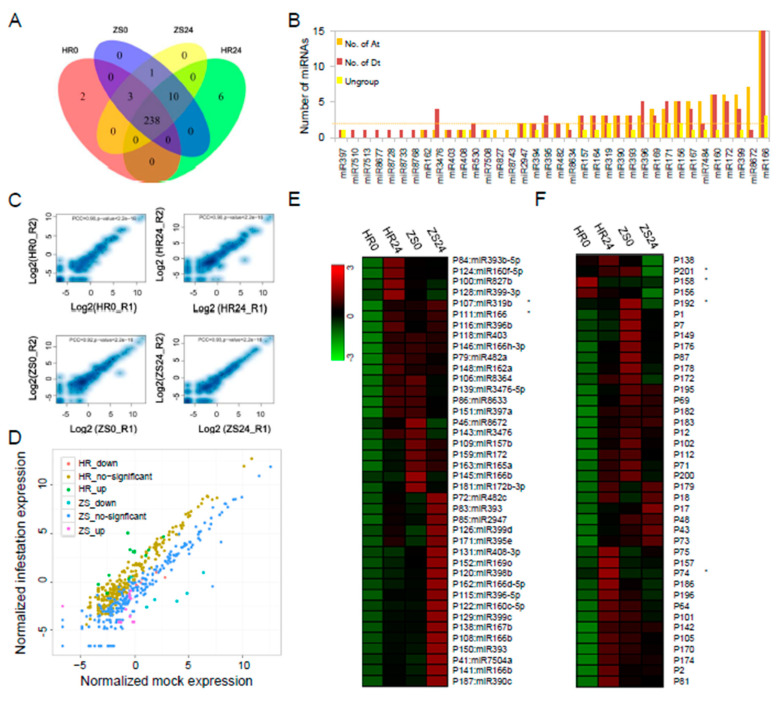
Comparative analysis of miRNAs and their expression profiles between whitefly-resistant (HR) and whitefly-susceptible (ZS) cotton plants during whitefly infestation. (**A**) The number of miRNAs following whitefly infestation in the HR and ZS cultivars. (**B**) The number of conserved pre-miRNAs in the A_t_-, D_t_-subgenomes and ungroup (scaffold). (**C**) Expression level (RPM) correlation of allthe miRNAs calculated by PCC in two biological replicates. (**D**) Scatter-plot graph representing differential expression profile of miRNAs between the HR and ZS plants following whitefly infestation. (**E**,**F**) Heatmap based on the abundance expression profiles of conserved and novel miRNAs from each cotton type during mock and whitefly infestation (* indicates *p* < 0.05 and log2|(infestation/control) | > 1).

The identification of miRNAs involved in the HR and ZS dataset was assessed and visualized using a Venn diagram (Figure 2A). We also systematically studied the distribution of the 475 identified pre-miRNAs, the pre-miRNA gene loci in the A_t_- and D_t_-subgenomes were analysed using BLASTN (Figure 2B). Multiple precursor gene loci (ranging from 1 to 33) were identified for miR156/7, miR160, miR166/7, miR169, miR171/2, and miR396/miR399 in both the A_t_- and D_t_-subgenomes (Figure 2B). More copies of the miR7484 and miR8672 precursors were found in the A_t_-subgenome than in the D_t_-subgenome (5 *vs*.2 and 7 *vs*.1, respectively). This distribution was flipped for the miR3476 and miR396 precursors, with more copies in the D_t_-subgenome (1 *vs*.4 and 3 *vs*.5). All of the miRNA expression level correlations from the HR and ZS datasets (Figure 2C), which represent two biological replicates, were calculated by PCC (PCC = 0.9). We found that 83 miRNAs (31.9%) were abundantly expressed with reads count > 200. For example, the miR156/7, miR160, miR166, miR390, miR396, miR398, and miR482 families were highly expressed in both HR and ZS plants infested with whiteflies for 24 h (Table S3). Only eight miRNAs were significantly differentially expressed between the HR and ZS cultivars, including P107:miR319b, P191:miR319b, P74, P169, and P182 in HR and P33:gra-miR8672, P27, and P242 in ZS (Figure 2D and Table S3). Based on the DESeq analysis, the expression levels of 23.1% of the miRNAs (60 of 260) were up-regulated in response to whitefly infestation in HR plants at 24 h, but though most of these miRNAs were down-regulated in ZS (Figure 2E,F). 

### 2.2. Abundant lincRNA Act as miRNA Precursor

LincRNAs are endogenous ncRNAs that are transcribed from genome intergenic regions and may play critical roles in regulating gene expression through multiple RNA-mediated gene regulation mechanisms [28]. To study further the function of the lincRNA-miRNA-mRNA cascade in the cotton response to whitefly infestation, we used our previously generated RNA-Seq dataset to identify potentially important polyA-containing lincRNAs. A comprehensive bioinformatics pipeline, developed to facilitate the identification of lincRNAs based on RNA-Seq datasets, is showed in Figure S3 (also see Materials and Methods). In total, 2365 strand-specific lincRNA genes were identified and corresponded to 6651 lincRNA transcripts for further analysis (Tables S4 and S5). We aslo found that the genomic loci of 2365 lincRNAs overlapped with 475 predicted pre-miRNAs and that seven conserved miRNA precursors and 10 novel miRNA precursors were generated from 29 lincRNA transcripts (Table 2). 

The secondary structure of seven conserved miRNAs and their corresponding lincRNAs are shown in Figure S4. These results indicated the lincRNAs can serve as miRNA precursors that regulate downstream target genes.

### 2.3. Analysis of the miRNA Target Genes Based on Degradome Sequencing

To provide further insights into the putative miRNA targets, a mixed degradome library was constructed from the HR0, HR24, ZS0 and ZS24 mRNAs. Approximately 14 million high quality reads were mapped to the *G. hirsutum* transcriptome after removing the adaptors and poor sequences (Table S6). Using degradome sequencing, 241 mRNAs and 13 lincRNAs were identified as targets for 81 miRNAs (Table S7). These lincRNAs were cleaved by the following three conserved miRNAs: (1) miR167, (2) miR396, and (3) miR399 (Figure S5). As expected, 41 conserved miRNAs had multiple targets that included transcription factors (TFs) and R genes. Among the most conserved miRNAs and targets were miR156-SPL, miR160-ARF, miR164-NAC, and miR828-MYB. More importantly, several novel miRNAs and targets were also detected, such as miR482a-AP2/B3 TFs and miR482c-receptor like kinase (RLK) (Figure S6).

**Figure 3:**
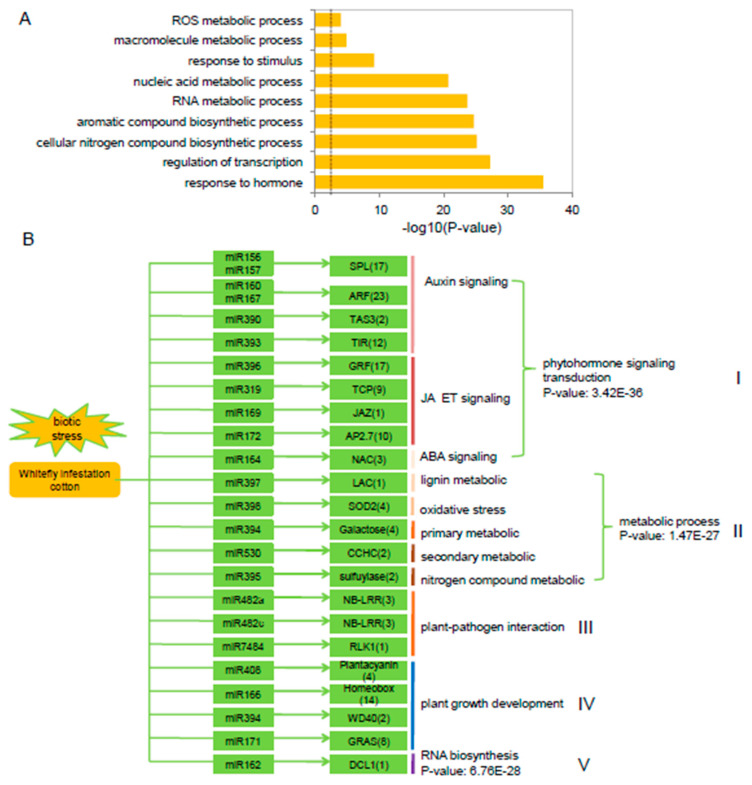
Validation of miRNA target genes based on degradome sequencing. (**A**) Gene ontology (GO) enrichment analysis of miRNA targets in cotton plants following whitefly infestation. The dashed line represents *p* < 0.01. (**B**) Overview of 24 conserved miRNA families, their corresponding targets and potentially associated pathways in cotton plants in response to whitefly infestation. *p*-values indicate the GO enrichment significant differences.

To better understand the regulatory roles of miRNAs in the transcriptional response of cotton to whitefly infestation, we also analyzed 241 targets for GO enrichment annotations. GO enrichment analysis revealed that ‘hormonal response genes’, ‘metabolic processes’, and ‘ROS metabolism-related genes’ were among the significantly enriched processes (*p* < 0.01) (Figure 3A). Constructing the cotton miRNA:mRNA interactome provided a visual platform to evaluate the induced/suppressed miRNA target gene networks in cotton in response to whitefly infestation (Figure 3B). Among the networks, 24 conserved miRNA target families were identified that were involved in phytohormone signal transduction, secondary metabolism, plant-pathogen interactions, plant growth development and RNA biosynthesis (Figure 3B). Based on the assignment of the functional category annotations, various relationships between miRNAs and their target genes were observed. (I) Five miRNAs, including miR156/7 with SPL, miR160/167 with ARF, and miR393 with the auxin signaling receptor F-box2 protein (TIR), are involved in regulating auxin signaling as well as auxin perception. miR390 is also involved in auxin signaling through the production of a tasiRNA that targeted ARFs [29]. Four miRNAs, including miR319 with TCP family transcription factor, miR169 with jasmonate-zim-domain protein (JAZ), miR396 with growth-regulating factor (GRF) and miR172 with AP2 TFs, are associated with JA and ET signaling pathways. miR164 is associated with *NAC100* and involved in the ABA signaling pathway. (II) miR397 is associated with a laccase (LAC) involved in lignin metabolism. miR398 with superoxide dismutase (SOD) and associated oxidative stress. miR394 with galactose and involved in primary metabolic, and miR530 with the zinc knuckle (CCHC-type) family protein and involved in secondary metabolism. (III) miR482 is associated with NB-LRR and miR7484 with a receptor-like protein kinase (RLK), both of which have roles in plant-pathogen interactions. (IV) miR166 is associated with a homeobox-leucine zipper protein and miR394 with a WD40, which are associated with plant growth and development. (V) miR162 is associated with *DCL1* and involved in RNA biological processes (Figure 3B). 

### 2.4. Tight Linkage Between miRNAs and Their Targets in Cotton in Response to Whitefly Infestation

**Figure 4:**
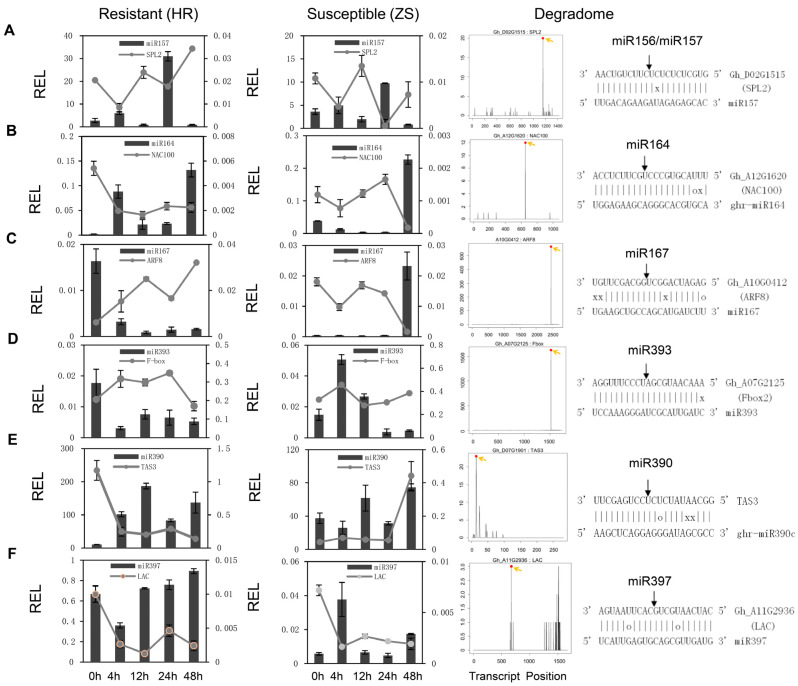
Expression profile of select miRNAs and their corresponding target genes in HR and ZS cotton plants following whitefly infestation. (**A**–**F**) The histograms and associated lines indicate the relative abundance of the individual miRNAs and their corresponding targets, respectively. The left and middle panels correspond to the expression profiles of the indicated transcripts in HR and ZS plants infested with whiteflies at different time points, respectively. The T-plots in the right panel show the relative abundance of the degradation signal mediated by the miRNAs. The associated miRNA:mRNA duplex alignment is shown above each panel. The G-U pairs are indicated with an ‘o’ and mismatch base pairs are indicated with an ‘x’.

To validate the miRNA expression patterns and their corresponding targets, six conserved miRNAs/targets were analyzed by stem-loop qRT-PCR in the HR and ZS plants (Figure 4). miR157 exhibited dynamic expression with abundance peaks at 4 and 24 h, whereas the expression profile of its corresponding target squamosa promoter binding protein-like 2 (*SPL2*) displayed the opposite, with decreased at those same time points (Figure 4A). Whitefly infestation promoted the up regulation of miR164 in HR plants, with a slight reduction in expression at 12 and 24 h. This contrasted with the miR164 expression profile in ZS plants, which was barely detectable until 48 h. The miR164 corresponding target *NAC100* was rapidly downregulated in the HR plants in accordance with increased miR164 expression and remained repressed throughout whitefly infestation (Figure 4B). However, in ZS plants, it peaked at 24 h when miR164 expression was non-detectable and was then drastically downregulated at 48 h as miR164 levels rose. miR167 and its target *ARF8* exhibited reciprocal expression profiles in HR and ZS plants with elevated miR167 levels during the initial stage and repressed levels during the later stages in HR plants, whereas expression was lowest at the early stages and highest (ca. 50-fold) at 48 h in ZS plants (Figure 4C). Similarly, the expression of miR393 in HR plants was lowest at 4 h of whitefly infestation but highest in ZS plants (Figure 4D). miR390/*TAS3* had a similar expression profile as miR157/*SPL2*, with expression in HR plants at all time points (Figure 4E). However, it showed down-regulation at the 4 and 24 h time points in the ZS plants. miR397 also exhibited reciprocal expression with periods of low expression in HR plants that corresponded to elevated expression in ZS plants (Figure 4F). Most importantly, target gene expression was negatively correlated with miRNA expression, suggesting that the miRNAs repress the expression of their target genes in response to whitefly infestation (Pearson’s correlation coefficient, *R*^2^ = −0.323, *p* = 0.00554, Figure S7).

### 2.5. Identification of miRNA-mediated phasiRNAs during the Whitefly Infestation Cotton Plants

**Figure 5:**
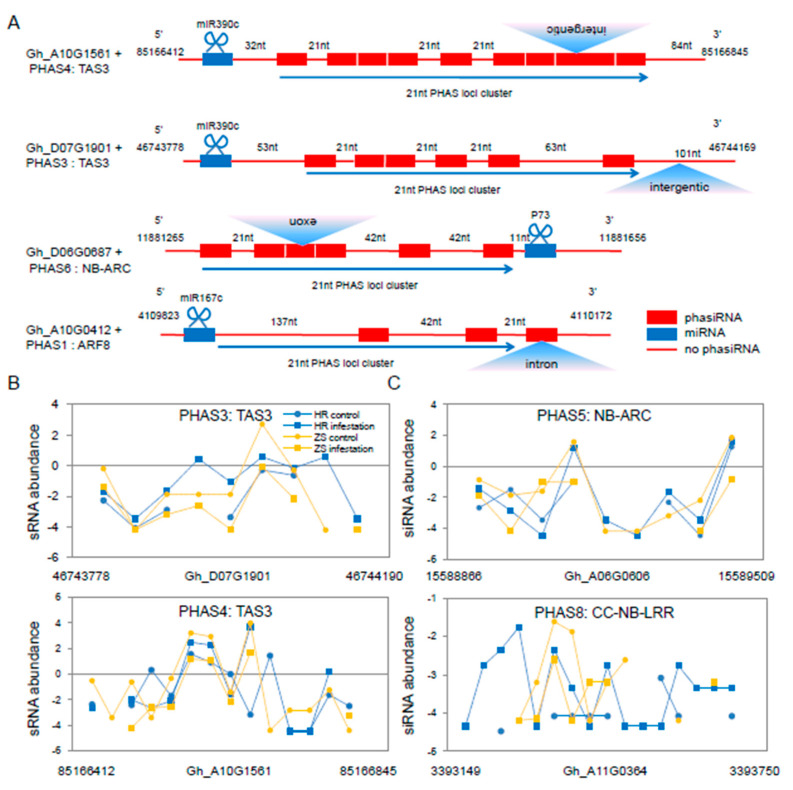
Characteristics of select siRNAs and various *PHAS* loci in cotton following whitefly infestation. (**A**) Schematic diagram of the 21-nt siRNAs generated from the *PHAS* loci in cotton following whitefly infestation. The structures of three typical *PHAS* genes are showed with phasiRNAs denoted by red boxes (indicating different siRNA sequences). Blue boxes represent the miRNA cleavage sites and red lines indicate non-*PHAS* loci. Blue triangles represent gene locations. (**B**) The expression profile of siRNAs in *TAS3*. (**C**) The *PHAS* siRNA derived from the NB-LRR disease-resistant protein sequence.

We identified 402 *PHAS* loci with 33 loci located in mRNAs (Table S8). In total, 85 siRNAs were generated from nine *PHAS* genes through six miRNA-mediated processes (Table S9). Several of the most conserved *PHAS* loci identified included those encoding two *TAS3*, two *ARF8*, four NB-LRR, and one AP2/B3, proteins (Figure S8). We found that two miR482 members and two novel miRNAs were 22nt in length. Interestingly, the two novel miRNAs (P73, and P81) triggered phasiRNAs from the *NB-LRR* gene sequence. The *PHAS* loci are located in diverse regions of the cotton genome including in exon, exon-intron, exon-intergenic and untranslated regions (Figure 5A and Figure S9). For example, the *PHAS5* and *PHAS8* loci are located in exonic regions of *NB-LRR*. However, there was no significant difference in the number of phasiRNAs generated from the *PHAS3* and *PHAS4* loci between the HR and ZS plants, and most siRNA expression levels were up-regulated in HR plants and down-regulated in ZS at 24 h (Figure 5B). Although the expression of *PHAS5* and *PHAS8* were up-regulated in whitefly-infested ZS plants, these loci generated more than twice the number of siRNAs in infested HR plants (18 in HR *vs*. 8 in ZS, Figure 5C). 

### 2.6. mi482a-Triggered phasiRNAs Regulate the Transcriptional in Cotton Response to Whitefly Infestation

**Figure 6:**
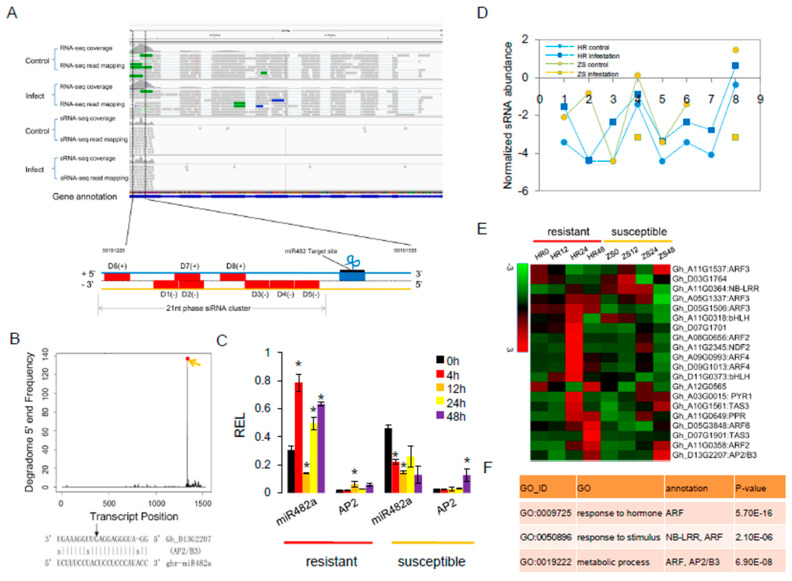
The miRNA-mediated phasiRNA pathway involved in the cotton response to whitefly infestation. (**A**) Integrative Genomics Viewer (IGV) depiction of miR482a-mediated cleavage of the AP2/B3 transcription factors and the resulting 21-nt phasiRNAs. The schematic diagram depicts the secondary siRNA biogenesis that is initiated at the 5′-target site. (**B**) Degradome data showing miRNA-guided target cleavage sites. (**C**) Expression profile of miR482a in HR and ZS plants following whitefly infestation. Three biological replicates were assayed for each control and the whitefly-infested samples (Student’s t-test *p < 0.05). (**D**) siRNA abundance profile from the phased region. (**E**) Expression profile of phasiRNA targets in HR and ZS plants following whitefly infestation. (**F**) GO enrichment analysis of phasiRNA target genes (*p* < 0.01).

We also found that miR482a targeted AP2/B3 and generated eight phasiRNAs. However, both RNA-Seq and sRNA-Seq data indicated that these phasiRNAs were not differentially expressed in HR plants in response to whitefly infestation at 24 h (Figure 6A). The AP2/B3 cleavage site that generated the highest abundance of phasiRNAs was confirmed by the degradome data (Figure 6B). The qRT-PCR analysis revealed a significant negative correlation between miR482a and AP2/B3 expression in both HR and ZS plants after whitefly infestation (Figure 6C). Furthermore, the abundance of phasiRNAs decreased in ZS plants after whitefly infestation (Figure 6D). To better understand the relationship between the nine *PHAS* genes and the 85 derived phasiRNA targets, twenty-eight mRNAs were identified as phasiRNA targets (Table S10). In addition to the well-known ARF family that is targeted by *TAS3* phasiRNAs, several phasiRNAs target genes related to biotic stress resistance were also identified including TFs (AP2/B3 and bHLH), an NB-LRR, a PPR protein, and an auxin-response factor. Overall, the expression of some of the target genes was up-regulated in HR plants but down-regulated in ZS plants within 24 h of whitefly infestation (Figure 6E). GO enrichment analysis also suggested that the most abundant phasiRNA targets function in plant responses to a hormone/stimulus and metabolic processes (Figure 6F).

### 2.7. Characterization of the linc1-miR390-tasiARFs Cascade Involved in Cotton Response to Whitefly Infestation

**Figure 7:**
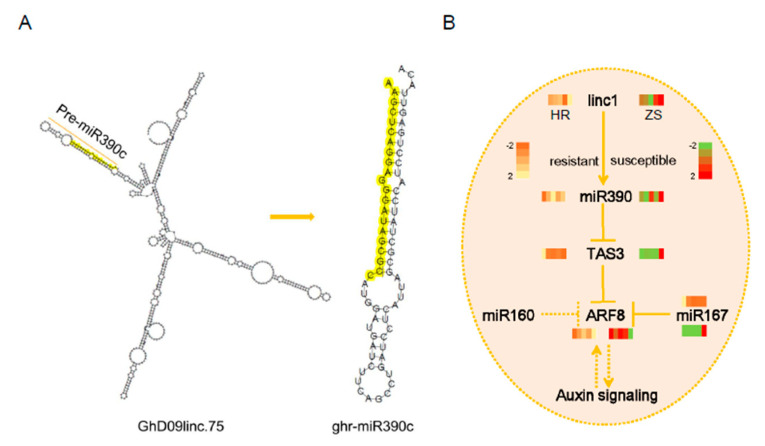
Proposed model of miR390-mediated tasiARFs. (**A**) The biogenesis of miR390 from *linc1*. (**B**) The expression patterns of gene involving in *miR390-tasiARF*s in cotton response to whitefly infestation.

*TAS3* tasiRNA biogenesis is triggered by miR390-directed cleavage of the *TAS3* transcript and has been reported previously [13,30,31]. Based on the bioinformatics and expression analyses, miR390 produced from precursor *linc1* (Figure 7A). We have proposed a model for miRNA-mediated plant development and host response to herbivore infestation (Figure 7B). *TAS3* and *ARF8* expression in HR and ZS plants exhibited contrasting patterns during whitefly infestation, which was mediated by miR390. These phasiRNAs regulate *AFR8* expression, which in turn influences the auxin signaling pathway affecting both plant development and host resistance. In addition to miR390, miR160 and miR167 also have moderate effects on *ARF8* expression suggesting that miRNAs have multiple target genes and exhibit diverse functions in host plant defense systems.

**Figure 8:**
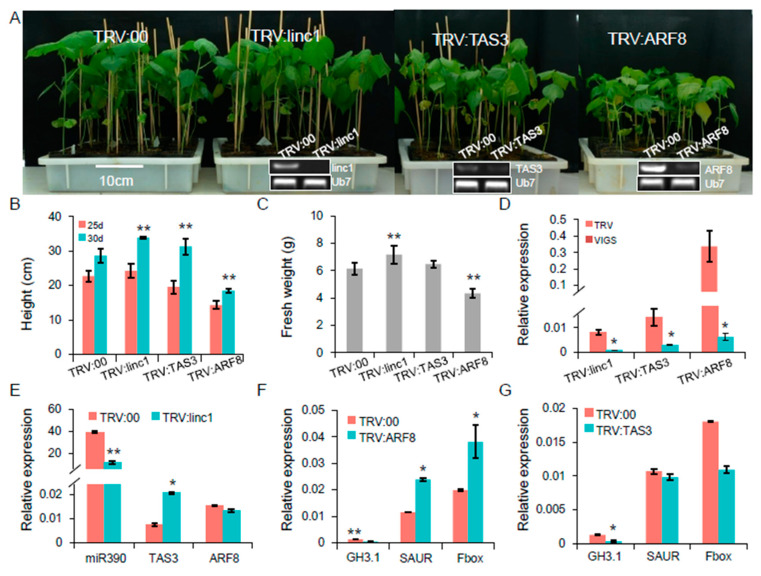
Functional of the miR390-mediated pathway using the virus-induced gene silencing (VIGS) technology. (**A**) Phenotype for *TRV:linc1*, *TRV:TAS3* and *TRV:ARF8* plants. At the bottom of the electrophoresis figure confirmed down-regulation of VIGS target genes in *TRV:linc1*, *TRV:TAS3* and *TRV:ARF8* plants compared with *TRV:00*. (**B**,**C**) Plant height and fresh weight on VIGS plants. (**D**) qRT-PCR analysis of *linc1*, *TAS3* and *ARF8* expression in different VIGS plants. (**E**) Silencing *linc1* leads to down-regulation of miR390 in *TRV*:*linc1* plants. (**F**,**G**) qRT-PCR analysis of auxin-related genes in *TRV:ARF8* and *TRV:TAS3* plants.

To further evaluate the functional characterization of this cascade, we investigated the functions of *linc1*, *TAS3,* and *ARF8* in plant growth and host resistance to whitefly infestation using VIGS (Figure S10). In this experiment, the albino phenotype of *TRV:GbCLA1* plants manifested ten days post-agroinfiltration was maintained for more than one month, suggesting that the VIGS system was efficient and durable. At three weeks post-agroinfiltration, plants with silenced target genes exhibited clear phenotypes. RT-PCR analysis confirmed that *linc1* expression levels were significantly reduced in the *TRV:linc1* plants compared to the *TRV:00* plants and that *TAS3* and *ARF8* expression levels were likewise dramatically decreased in the *TRV:TAS3* and *TRV:ARF8* plants (Figure 8A). The *TRV:ARF8* plants were significantly smaller (reduced height and fresh weight) than the control *TRV:00* (empty vector) plants (Figure 8B,C). qRT-PCR further confirmed that *linc1*, *TAS3*, and *ARF8* expression levels were significantly down-regulated (10–54 fold lower than the control) in most VIGS plants (Figure 8D). Silencing of *linc1* in the *TRV:linc1* plants resulted in the down-regulation of miR390 expression and up-regulation of *TAS3* (Figure 8E). We next analysed the expression of the following three major receptor proteins involved in auxin signaling in the *TRV:TAS3* and *TRV:ARF8* plants: (1) the auxin-responsive GH3 family protein *GH3.1* (*Gh_D02G2045*), (2) the SAUR auxin-responsive protein *SAUR* (*Gh_A13G1024*), and (3) the auxin F-box protein (*Gh_D05G2130*). However, there were no obvious changes in the expression of SAUR- and Fbox-encoding genes in the *TRV:TAS3* plants, while *GH3.1* was substantially down-regulated (Figure 8F).

**Figure 9:**
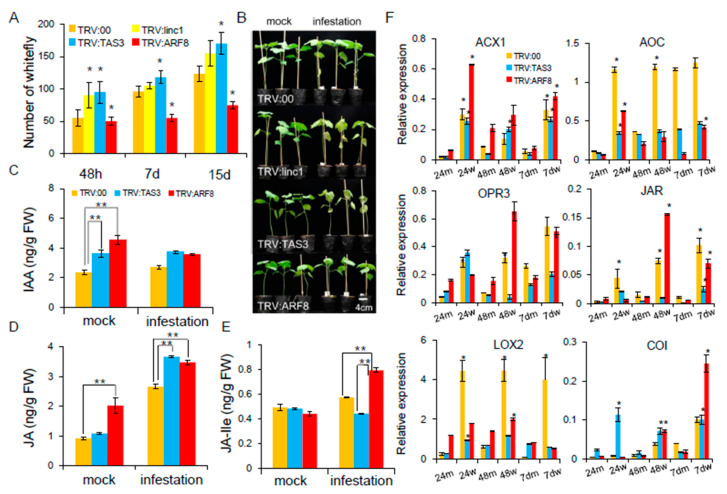
The auxin and JA signaling crosstalk miR390-*tasiARFs* involved in the cotton following whitefly infestation. (**A**) The number of whiteflies on VIGS plants. (**B**) The phenotypes of *TRV:00*, *TRV:TAS3*, *TRV:ARF8* after whitefly infestation cotton plants at two-weeks. (**C–E**) Jasmonic acid (JA) and indole-3-acetic acid (IAA) concentrations were measured by LC-MS in *TRV:TAS3* and *TRV:ARF8* plants. (**F**) qRT-PCR validation of JA biosynthesis related genes after whitefly infestation VIGS plants. Error bars indicate S.D from four biological replicates. The statistical test was performed for each mock and whitefly-infested sample in in *TRV:00*, *TRV:TAS3* and *TRV:ARF8* (Student’s *t*-test *p < 0.05). The 24m, 48m, 7dm, 24w, 48w, and 7dw represent mock and whitefly infestation cotton at 24 h, 48 h, 7 days, respectively.

The *TRV:TAS3* and *TRV:ARF8* plants were selected for further study on the basis of higher insect resistance. Whitefly colonization of *TRV:ARF8* plants was moderately reduced at two weeks post-infestation, whereas there was no significant difference in whitefly densities on the *TRV:linc1* and *TRV:TAS3* plants (Figure 9A). Whitefly feeding on the *TRV:TAS3* and *TRV:00* plants caused chlorosis and drooping of the leaves in the greenhouse, whereas the *TRV:ARF8* plant showed normal growth apart from plant height (Figure 9B). Analysis of the signaling hormones revealed that auxin levels were reduced in the *TRV:ARF8* plants following whitefly infestation (Figure 9C). JA plays a role in plant-herbivore interactions [20]. These results indicate that the enhanced resistance to whiteflies observed in the *TRV:ARF8* plants is likely due to combinatorial effects that arise from the suppression of auxin signaling pathway and induction of JA signaling (Figure 9D). Jasmonoyl-l-isoleucine (JA-Ile) levels, which are catalyzed by JAR enzymes from JA, were examined. The *TRV:ARF8* plants accumulated higher JA-Ile levels compared with *TRV:00* plants after whitefly infestation (Figure 9E). The expression profiles for genes involved in the JA signaling pathway were measured in cotton plants infested by whitefly. qRT-PCR showed that acyl-CoA oxidase 1 (*ACX1*), allene oxide cyclase (AOC), *LOX2*, oxophytodienoate-reductase 3 (*OPR3*), JAR enzymes (JAR), and COI receptor (COI) expression levels were more rapidly induced in *TRV:ARF8* than *TRV:00* (Figure 9F). These data suggest that JA biosynthesis may be partially induced in *TRV:ARF8* plants by whitefly infestation.

## 3. Discussion

Non-coding RNAs play essential roles in plant development and stress signaling transduction pathways [10,12]. While the application of RNA-Seq methodologies to analyze the expression of ncRNA responses to biotic stresses in model plants is well-established, similar studies in cotton and reports linking changes in ncRNA abundance with plant-herbivore interactions are more limited [23,25,32,33,34]. Despite the widespread use of traditional chemical-based strategies for whitefly control in cotton, epidemics remain prevalent worldwide [35]. Consequently, elucidating the molecular mechanisms underlying cotton host resistance to whitefly infestation is highly desirable. RNA-Seq analysis has allowed us to characterize lincRNA dynamics during whitefly infestation in cotton, with more detail that in previous studies in *G. barbadense* [36]. lincRNAs act as competing for endogenous RNAs that bind to special miRNAs as target mimicsto protect target mRNAs from degradation in plants [37]. We predicted a similar mimetic relationship between lincRNAs and miRNAs with 13 cotton lincRNAs likely to act as decoys for miRNAs, such as miR160, miR167, miR399, the miR482 family, and six novel miRNAs. 

In addition to their role as miRNA target mimics, abundant lincRNAs may be degraded into pre-miRNAs. Previous studies have evaluated the expression of different precursor genes for miR156/7, miR164, miR171/2 and miR396 in tomato fruit development and ripening [38]. However, the detailed functions of these miRNAs remain unknown and require further study through the overexpression of eTMs and miRNAs. The expression profile of lincRNAs, their corresponding miRNAs, and their downstream targets suggest that their functions are closely interrelated. Furthermore, miR156, miR390, and the miR482 family were found to negatively regulate *SPL2*, *TAS3,* and AP2/NB-LRR, respectively, consistent with previous reports on the transcriptional response of cotton to disease. However, contrasting miR482a expression profiles were observed in whitefly-infested resistant and susceptible plants, suggesting that it may function in both plant host resistance and herbivore infestation. As expected, trends observed in the expression of lincRNAs were consistent with their corresponding miRNAs, suggesting that lincRNA-miRNA cascades may play important roles in cotton in response to whitefly infestation.

Previous studies reported that miRNA-triggered phasiRNAs play a major role in plant-pathogen interactions [39]. To elucidate the potential function of phasiRNAs in plant-insect interactions, we evaluated phasiRNA targets and conclude that they may have plant hormone-related functions. Genomic structure analysis revealed that five 22nt miRNAs that target R genes contained multiple exons and various siRNA production loci (Figure 5A). More importantly, transcriptional profiling showed that these phasiRNAs are in low abundance, with fewer loci in the whitefly-susceptible ZS plants. Similar results were observed in the tomato transcriptional response to tobacco mosaic virus (TMV) infection [40]. We also identified a new miR482a-targeted gene, AP2/B3. The expression of AP2/B3 did not dramatically change, though the expression of the phasiRNAs were downregulated and the phasiRNAs were diminished after whitefly infestation. These results suggest that cascades of lincRNAs, miRNAs, *PHAS* and their targets are fundamental in plant pathways activated in response to herbivore infestation. 

Diverse miRNA/siRNA-mediated R genes predicted to regulate phytohormone signaling were implicated in cotton in response to herbivory. This finding is not surprising, as miRNA targets associated with phytohormone signaling (e.g., JA, ET, and auxin) have been reported following herbivore infestation in *Nicotiana attenuate* and *Cucumis melo* [24,25]. Silencing the *RDR1* gene in *Nicotiana attenuata* increased plant susceptibility to insect herbivory, suggesting that this defense system may be regulated by sRNAs [24]. Auxin has been recognized as a positive regulator of the plant defense system. It has also been shown to have roles in microbial-induced disease resistance [41]. As shown in Figure 3B, most miRNA target genes negatively regulated by miRNAs were associated with phytohormone signaling. Notably, both miR393 and miR167 negatively regulated *ARF8* during whitefly infestation and then further affected the auxin signaling pathway involved in cotton plant development and insect resistance. A subset of these tasiRNAs plays important roles in auxin response through ARF repression and by regulating the expression of specific target genes [42]. Moreover, miR390-mediated *TAS3* negatively regulated *ARF8* expression at all time points and *ARF8* expression showed opposite patterns in the resistant and susceptible plants during whitefly infestation. Previous miRNA profiling studies demonstrated that the activation of miR393 and miR167 resulted in auxin insensitivity during aphid infestation in resistant *Cucumis melo* [25]. The previous report the miR156/SPLs module function regulates developmental and resistance [43]. miR390, which is produced from a *linc1* precursor, binds *TAS3* to generate eight phasiRNAs that regulate *ARF8* expression, which in turn influences the auxin signaling pathway by affecting both plant development and host resistance. In addition to miR390, miR160 and miR167 also have moderate effects on *ARF8* expression, suggesting that miRNAs have multiple target genes and exhibit diverse functions in host plant defense systems. 

## 4. Materials and Methods

### 4.1. Plant Materials, Whitefly Infestation, and RNA Isolation

*G. hirsutum* seeds from the whitefly-resistant cultivar (HR) and a susceptible cultivar (ZS) were germinated on 1/2 Murashige and Skoog (MS) medium [26]. After germination, the seeds were maintained in the dark for two days. Plantlets were then grown for five days at 28 ± 2 °C and a 16 h/8 h day light/dark cycle until they developed flat cotyledons. Whiteflies (*Bemisia tabaci*) were fed on potted cotton plants at 28 ± 2 °C and 70% relative humidity in the greenhouse. Adult whiteflies were collected from the greenhouse by aspiration into Falcon tubes, and fifty adults were transferred to cotton plantlets with two flat cotyledons. Adult whiteflies (50 whiteflies) were removed from the cotton plants 24 h after infestation. Mock plant cotyledons (24 h) were grown in boxes without whitefly infestation. Total RNA was isolated using a modified guanidine thiocyanate method [44].

### 4.2. Small RNA and Degradome Library Construction

The quality of total RNA was assessed using an Agilent 2100 Bioanalyzer (Agilent, CA, USA). A single TruSeq Small RNA Sample Prep workflow is summarized as follows. Briefly, 1 μg total RNA was ligated to 5′- and 3′-RNA adaptors and reverse transcribed into cDNA. After PCR amplification, libraries from separate samples were pooled for 15% polyacrylamide gel isolation. In total, twelve RNA libraries were constructed, consisting of three biological replicates corresponding to the HR and ZS plants infested with whiteflies for 0 h and 24 h. The cDNA pools were sequenced on an Illumina Genome Analyzer (San Diego, CA, USA) at the National Key Laboratory of Crop Genetic Improvement in Huazhong Agricultural University (Wuhan, China). Each treatment RNA samples (including two biological replicates) were pooled (20 μg) and one degradome library was constructed as previously described with minor modifications [45]. The ligation products were amplified and sequenced on an Illumina Genome Analyzer.

### 4.3. miRNA Prediction Pipeline

The raw reads were pre-processed with the NGSQC toolkit [46] to remove the low-quality reads (quality score, Q < 20, reads shorter than 18nt) and trim the adaptor sequences. Reads were mapped to the Rfam database to exclude snRNAs, snoRNAs, tRNAs, and rRNAs. The remaining sRNAs were subjected to miRNA identification. The final clean reads were mapped to the *G. hirsutum* genome [47] using bowtie (-v 0 -m 200). We used structure- and probability-based annotation to predict the miRNA loci as described previously [48]. For the structure-based annotation, sequences 150-bp upstream and downstream of the miRNA mapping sites were extracted and defined as “pre-miRNA putative sequences”. miRcheck was used to evaluate the RNA secondary structures, and hairpin-like structures were predicted using RNAfold [49]. For the probability-based annotation, the putative precursors from the structure-based annotation were filtered using miRDP [50]. The cutoff value of the largest miRNA family size was set at 50 because of the triple genome size of the tetraploid cotton compared to diploid cotton. All of the annotated mature miRNAs were searched against miRBase21 [51] and categorized into cotton conserved and novel miRNAs families using the BLASTN program. 

### 4.4. Expression Profiles of miRNAs in the HR and ZS Plants during Whitefly Infestation

The expression levels of all the miRNAs and siRNAs were normalized into reads per million (RPM) [RPM = miRNA reads*10^6^/ total reads]. DESeq was used to evaluate the miRNA differential expression analysis in the HR and ZS cultivars with the following criteria: (1) |log2(treatment/control) | > 1 and (2) an adjusted *p*-value < 0.05 [52].

### 4.5. Identification and Functional Annotation of the miRNA Target Genes 

The CleaveLand pipeline (*p* < 0.05) was used to detect putative miRNA targets [53]. The miRNA targets were predicted by *G. hirsutum* transcriptome [47] and our identified lincRNA datasets. Candidate targets were then categorized (between 0 and 4) based on the relative abundance of the miRNA/siRNA-mediated cleavage site compared to the total number of tags. Gene ontology (GO) analysis was performed using the Blast2GO software with a false discovery rate (FDR) < 0.05 based on Fisher’s exact test.

### 4.6. Identification of lincRNAs from RNA-Seq Dataset

Our recent transcriptome data examining the response of cotton to whitefly infestation [26] were assembled using Cufflinks 2.0 according to the provided instructions [54]. Briefly, each RNA-Seq dataset was independently aligned to the *G. hirsutum* genome using TopHat 2.0 [55]. Then, all the transcriptome data were pooled and merged to generate a final transcriptome using Cuffmerge. After the final transcriptome was generated, Cuffdiff was used to estimate the abundance of the transcripts based on the final transcriptome, and a BAM file was generated from the TopHat alignment. Cuffcompare was used to annotate novel transcripts (class_code = “u”) against the known annotation. We discarded transcripts that overlapped either 500-bp upstream or downstream of mRNAs, that had FPKM value < 0.5 and were shorter than 200-bp. The novel transcripts were analyzed against the Pfam and Swiss-prot databases using BLASTX (e-value < 1*e*^−3^). The remaining transcripts were then subjected to coding-non-coding index (CNCI) analysis to exclude specific transcripts, and the remaining transcripts were reliably defined as expressed lincRNAs [56].

### 4.7. Stem-loop qRT-PCR Analysis of miRNAs

To quantify the identified miRNAs and mRNAs, stem-loop RT-PCR was performed using a protocol with minor modifications [57]. All the qRT-PCR templates were generated from 3 μg total RNA isolated from the HR and ZS plants at 0, 4, 12, 24, and 48 h after infestation. The stem-loop PCR system (20 µl mixture) consisted of 0.05 µM stem-loop primers, 2.5 µM oligo-dT primer, 0.4 mM dNTPs, 4 µL 5×First-strand buffer, 1 µL DTT (100 mM), 1 μL RNase inhibitor, and 1 µL reverse transcriptase (Invitrogen, Carlsbad, CA, USA). The reverse-transcription reaction was performed with the following conditions: (1) 16 °C for 30 min, (2) 60 cycles of 30 °C for 30 s, 42 °C for 30 s, and 50 °C for 1 s, and (3) 85 °C for 5 min. Quantitative RT-PCR was performed as previously described [26]. Relative quantification of miRNA gene expression was calculated using *GhUBQ7* (GenBank accession number: DQ116441) as an internal standard. The comparative Ct method (2^−ΔΔCt^) was used to calculate transcript expression levels [58]. All the primers used in the qRT-PCR analyses are listed in Table S1. 

### 4.8. Identification of PHAS Loci

Genome-wide identification of phasiRNA loci was performed as described using PhaseTank (max_hits 30, number 4) [59]. The algorithm for the introduction of relatively small RNA production (RSRP) was adopted to reduce false phasiRNA prediction. The Integrative Genomics Viewer (IGV) was used to evaluate both the *PHAS* loci and associated expression levels [60].

### 4.9. Validation of lincRNA Function and Corresponding Targets by VIGS

Virus-induced gene silencing (VIGS) was performed as described [26]. Sequences for *linc1*, *TAS3*, and *ARF8* were amplified using primers listed in Table S1. The fragments were then cloned into tobacco rattle virus (TRV) vectors. The *TRV:linc1*, *TRV:TAS3*, *TRV:ARF8*, *TRV:00* (empty vector) and *TRV:CLA* (chloroplasts alterados) vectors were transformed into *Agrobacterium* GV3101 by electroporation. Agro-infiltration was performed using a syringe on 10-day-old *G. hirsutum* cv. HR seedlings. After two weeks, the *TRV:CLA* plants with an albino phenotype and other plants were used for further analysis. The analysis of the whitefly populations in the tested cotton plants in each experiment was assessed at 48 h, 7 days, and 15 days post-agroinfiltration. Approximately 0.1 g leaf samples were homogenized in 1 mL of 80% methanol and shaken overnight at 4 °C. Shaken samples were centrifuged at 12,000 rpm for 10 min and transferred to a 2 mL tube. IAA and JA extraction, purification, and quantification were performed with five biological replicates according to a method described previously with slight modifications [61].

### 4.10. Data Availability

The RNA-Seq, sRNA-Seq and degradome data generated in this report have been submitted to NCBI as a sequence read archive (SRA project: PRJNA286935).

## 5. Conclusions

To facilitate a better understanding of the role of ncRNAs in cotton-herbivore interaction, we developed a comprehensive framework for the genome-wide identification of ncRNAs in resistant and susceptible cotton cultivars following whitefly infestation. The 2365 lincRNAs, 260 miRNAs, 85 phasiRNAs, 241 miRNA targets were identified and found that the ncRNAs exhibited complex connections during herbivore infestation. We also used bioinformatics analyses and VIGS method to provide new insights into the miR390-*tasiARFs* cascade and propose that miRNA-mediated phasiRNA is a mechanism for the continuous regulation of downstream targets during plant responses to insect attack. In the current report, VIGS of *linc1*, *TAS3* and *ARF8* suggests the following conclusions: (1) *ARF8* might regulate gene expression in both the auxin and JA signaling, (2) down-regulating *linc1* leads to the down-regulation of miR390, and (3) silencing *ARF8* led to the accumulation of JA levels, through the induction of JA biosynthesis-related gene expression. *ARF8* and its regulatory miRNAs play a balancing role in cotton growth development and resistance, but the mechanism of which is not clear. Further analyses into the mechanism underlying miR390 regulation will require CRISPR/Cas9-mediated mutations to *linc1*, *TAS3* and/or *ARF8*. Only then will it be possible to demonstrate unequivocally the role of miRNAs in plant-insect interactions.

## Figures and Tables

**Table 1:** sRNA-Seq classification of twelve libraries.

Sample	Raw Reads	Unique Reads	SnoRNA	snRNA	5S_rRNA	sRNA	Mapping
HR0_R1	16,906,187	2,276,889	567	333	6957	977,245	84.19%
HR0_R2	21,306,455	2,281,508	949	554	10,990	600,015	83.81%
HR0_R3	11,613,784	729,752	213	227	2721	650,351	84.12%
HR24_R1	20,313,659	2,350,845	548	394	7678	824,200	81.82%
HR24_R2	22,215,560	2,404,771	184	93	1669	1,476,308	82.16%
HR24_R3	11,306,605	833,843	245	262	2932	745,572	84.45%
ZS0_R1	18,328,323	1,838,613	97	43	770	1,047,368	79.65%
ZS0_R2	21,173,195	2,457,802	149	68	1480	1,476,875	79.93%
ZS0_R3	11,335,617	1,086,277	232	245	2977	986,067	84.21%
ZS24_R1	18,240,711	1,104,283	70	44	940	636,559	81.41%
ZS24_R2	17,720,064	1,837,592	108	69	1051	507,170	82.14%
ZS24_R3	11,416,722	1,037,485	220	232	3176	925,213	83.51%

**Table 2:** The pre-miRNAs generated from lincRNAs in HR and ZS plants following whitefly infestation.

MiRNA_ID	miRBase21	Strand	Chr	Start	End	lincRNA_ID	miRNA Target Annotation
P132	osa-miR171f-3p	+	A05	15999334	15999553	GhA05linc.520	GRAS
P147	ghr-miR166b	+	A07	28328142	28328522	GhA07linc.319	Homeobox-leucine zipper
P147	ghr-miR166b	+	A08	8999568	8999969	GhA08linc.292	Homeobox-leucine zipper
P147	ghr-miR166b	+	A11	67055538	67055606	GhA11linc.93	Homeobox-leucine zipper
P149	NoHits	+	A08	103041407	103042061	GhA08linc.135	
P168	gra-miR8733	-	D06	692582	692872	GhD06linc.129	
P181	ath-miR172b-3p	-	A05	9079609	9079903	GhA05linc.451 (linc6)	related to AP2.7
P187	ghr-miR390c	+	D09	43446164	43448093	GhD09linc.75 (linc1)	TAS3
P193	ghr-miR156d	+	A07	2556261	2556647	GhA07linc.14	SPL
P72	gra-miR482c	+	A07	9733996	9734022	GhA07linc.38 (linc4)	NB-ARC
P73	NoHits	+	D05	43103308	43103635	GhD05linc.279 (linc5)	NB-ARC
P73	NoHits	-	D05	43102918	43103615	GhD05linc.670 (linc2)	NB-ARC
P81	NoHits	+	D05	43103308	43103635	GhD05linc.279	NB-ARC
P81	NoHits	-	D05	43102918	43103615	GhD05linc.670	NB-ARC
P87	NoHits	+	A12	84153544	84153795	GhA12linc.146	

## Supplementary Materials

The following are available online at https://www.mdpi.com/1422-0067/20/21/5357/s1, Figure S1: The phenotype during the whitefly infestation resistant and susceptible cotton cultivar. (A) The phenotype of HR and ZS cultivar after infestation 1 month. (B) The cotton plants infested by whitefly in a sealed chamber box; Figure S2: Distribution of sRNA reads in twelve libraries. (A) Distribution of total read lengths present in the three biological replicates. (B) AllsRNA (miRNAs+siRNAs) expression level correlations were calculated by PCC in the two biological replicates; Figure S3: Schematic diagram of the integrative pipeline used for systematic identification of lincRNAs; Figure S4: Conserved miRNA precursors generated by lincRNAs; Figure S5: T-plots of the cotton miRNA stargeted by lincRNAs. Figure S6: miR482 targets were confirmed by degradome sequencing; Figure S7: The expression of correlation between miRNA and their target gene; Figure S8: PHAS genes triggered by miRNAs identified in this report; Figure S9: PhasiRNAs generated from different cotton genome regions. ANB-LRR protein generated phasiRNA loci in two alternative exons by a novel P73 miRNA trigger. B Deg P protease-generated siRNAs from exon-intron-exon junctions. CNB-LRR protein generated siRNAs from a bona fide intron. D lincRNA-generated phasiRNAs; Figure S10: The VIGS vector construction; Table S1: Primers used in this study; Table S2: Expression level of all miRNAs in HR and ZS plants following whitefly infestation; Table S3: All of the pre-miRNA loci in the cotton genome; Table S4: All of the lincRNA loci in the cotton genome; Table S5: Expression profile of strand orientation lincRNAs; Table S6: Summary of degradome sequencing; Table S7: Validation of miRNA targets identified in degradome sequencing; Table S8: Identification of cotton PHAS loci in response to whitefly infestation; Table S9: miRNA-mediated phasiRNA expression. The blank region represents phasiRNAs that were lost; Table S10. Expression profile of phasiRNA targets in cotton following whitefly infestation.
