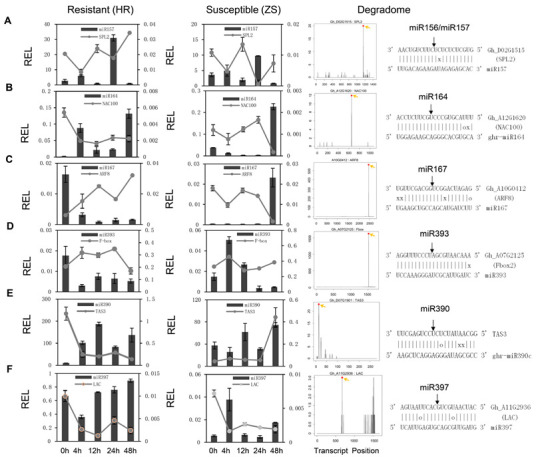# Correction: Li et al. Genome-Wide Analysis of Cotton miRNAs During Whitefly Infestation Offers New Insights into Plant-Herbivore Interaction. *Int. J. Mol. Sci.* 2019, *20,* 5357

**DOI:** 10.3390/ijms26093929

**Published:** 2025-04-22

**Authors:** Jianying Li, J. Joe Hull, Sijia Liang, Qiongqiong Wang, Luo Chen, Qinghua Zhang, Maojun Wang, Shahid Mansoor, Xianlong Zhang, Shuangxia Jin

**Affiliations:** 1 National Key Laboratory of Crop Genetic Improvement, Huazhong Agricultural University, Wuhan 430070, China; lijy90@126.com (J.L.); sijialiang@webmail.hzau.edu.cn (S.L.); wangqq0515@163.com (Q.W.); chenluo@webmail.hzau.edu.cn (L.C.); qhzhang@outlook.com (Q.Z.); mjwang@mail.hzau.edu.cn (M.W.);; 2 USDA-ARS, Arid Land Agricultural Research Center, 21881 North Cardon Lane, Maricopa, AZ 85138, USA; joe.hull@ars.usda.gov; 3 National Institute for Biotechnology and Genetic Engineering (NIBGE), Faisalabad 38000, Pakistan

In the original publication [1], there was a mistake in Figure 4B,E as published. The degradome sequencing results in Figure 4B,E were duplicated during the manuscript proofing process. The corrected Figure 4B appears below. With this modification, the Figure citation order is the same as before. The authors state that the scientific conclusions are unaffected. This correction was approved by the Academic Editor. The original publication has also been updated.